# The Role of Curcumin in Cancer Treatment

**DOI:** 10.3390/biomedicines9091086

**Published:** 2021-08-26

**Authors:** Vasiliki Zoi, Vasiliki Galani, Georgios D. Lianos, Spyridon Voulgaris, Athanasios P. Kyritsis, George A. Alexiou

**Affiliations:** 1 Neurosurgical Institute, School of Medicine, University of Ioannina, 45500 Ioannina, Greece; v.zoi@uoi.gr (V.Z.); svoulgar@uoi.gr (S.V.); thkyrits@uoi.gr (A.P.K.); 2 Department of Anatomy Histology-Embryology, School of Medicine, University of Ioannina, 45500 Ioannina, Greece; vgalani@uoi.gr; 3 Department of Surgery, University Hospital of Ioannina, 45500 Ioannina, Greece; georgiolianos@yahoo.gr; 4 Department of Neurosurgery, School of Medicine Ioannina, University of Ioannina, 45500 Ioannina, Greece

**Keywords:** curcumin, cancer, cell signaling pathways

## Abstract

Curcumin is a polyphenol extracted from the rhizomes of the turmeric plant, *Curcuma longa* which has anti-inflammatory, and anticancer properties. Chronic inflammation is associated with the development of cancer. Curcumin acts on the regulation of various immune modulators, including cytokines, cyclooxygenase-2 (COX-2), and reactive oxygen species (ROS), which partly explains its anticancer effects. It also takes part in the downregulation of growth factors, protein kinases, oncogenic molecules and various signaling pathways, such as nuclear factor kappa-light-chain-enhancer of activated B cells (NF-κB), c-Jun N-terminal kinase (JNK) and signal transducer and activator of transcription 3 (STAT3) signaling. Clinical trials of curcumin have been completed or are ongoing for various types of cancer. This review presents the molecular mechanisms of curcumin in different types of cancer and the evidence from the most recent clinical trials.

## 1. Introduction

**Figure 1:**
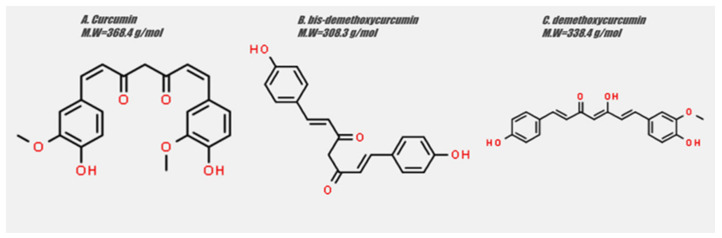
Chemical structure of (**A**) curcumin, (**B**) bis-demethoxycurcumin, (**C**) demethoxycurcumin. Structures were drawn using ChemSpider, an online free chemical structure database.

*Curcuma longa*, commonly known as turmeric, is a herbaceous plant belonging to the ginger family. [1] The plant produces a variety of secondary metabolites including flavonoids, alkaloids, tannins and phenolic acids [2], among which the active hydrophobic polyphenol diferuloymethane, named curcumin, is of special notice [3]. Curcumin is used in the treatment of various health conditions, including inflammatory disorders, liver disease, metabolic syndrome, neurodegenerative diseases and, most importantly, in several types of cancer [4]. The chemical structure of curcumin, as well as the most important curcuminoids, namely demethoxycurcumin and bis-demethoxycurcumin, are presented in Figure 1. 

**Figure 2:**
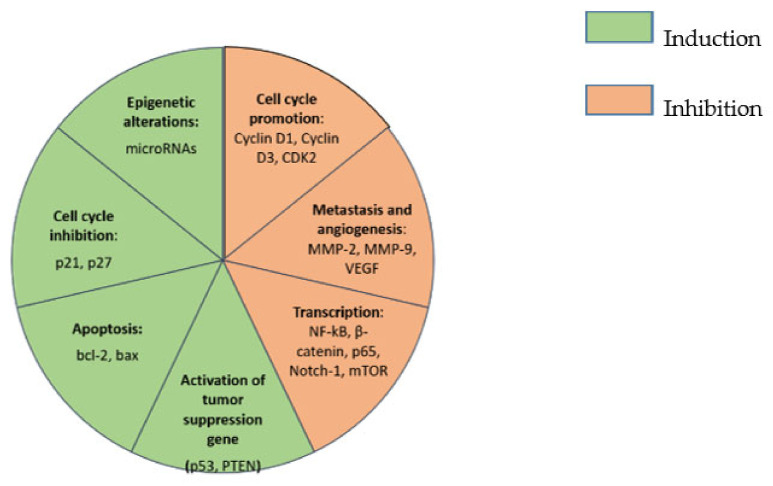
Summarized effects of curcumin on cancer cells. Key: CDK2, cyclin-dependent kinase 2; MMP-2, matrix metallopeptidase 2; MMP-9, matrix metallopeptidase 9; VEGF, vascular endothelial growth factor; NF-kb, nuclear factor kappa-light-chain-enhancer of activated B cells; mTOR, mechanistic target of rapamycin; PTEN, phosphatase and tensin homolog; bcl-2, B-cell lymphoma-2; bax, Bcl-2-associated X protein.

Cancer is among the primary causes of death worldwide [5] and, despite the high level of global awareness and the development of multitargeted therapeutic options, death rates from cancer are still significantly high [6]. Cancer cells are characterized by disruption of several signaling pathways including those responsible for angiogenesis, proliferation, metabolism, migration, immune modulation and survival [7]. Curcumin has been found to affect cancer cells in a variety of ways leading to the prevention of cancer formation. Its most prominent effects on cancer are summarized in Figure 2.

Curcumin has shown promising results in the treatment of several types of cancer both alone and in combination with other antineoplastic agents. It affects several signaling pathways and can thus effectively modify both the development and the growth of various tumors. This review summarizes the immunomodulatory effects of curcumin and the most recent evidence on the effectiveness of curcumin in the treatment of different types of cancer in vitro both alone and in combination with other chemotherapeutic agents.

## 2. Immunomodulatory Effects of Curcumin

Chronic inflammation disorders and infectious diseases are responsible for the development of several types of cancer [8], contributing to genomic instability, which is considered a hallmark of cancer [9]. The inflammatory process results in the production of several pro-inflammatory molecules, including reactive oxygen species (ROS), nuclear factor kappa-light-chain-enhancer of activated B cells (NF-Κb), cytokines, AKT, the transcription factor activator protein-1 (AP-1), and cyclooxygenase-2 (COX-2), which are involved in both the initiation and the growth of tumors [10]. Curcumin acts on the regulation of different immune modulators, resulting in some of its anticancer properties. 

ROS are molecules derived from oxygen with the ability to act as secondary messengers in several cellular signaling pathways. They participate in inflammation and in cell survival, differentiation and proliferation, resulting in the progress of different types of cancer [11]. Curcumin binds directly to ROS scavengers and may thus suppress the growth and metastasis of some types of cancer [12].

NF-κB factor regulates and controls the expression of various proteins, including cytokines and interferons. Those proteins are closely related to both inflammation and cancer progression [13]. Curcumin has an inhibitory action on the NF-κB-dependent pathway, resulting in the suppression of tumors and induction of apoptosis [14,15]. Cytokines are important regulators of the immune system that allow cells to communicate over short distances, and they control the proliferation, survival, differentiation, and apoptosis of leucocytes. In recent studies, cytokines, such as interleukin (IL) IL-12, IL-15 and interferon-a (IFN)-a, have been investigated in murine cancer models [16,17]. The expression of pro-inflammatory cytokines has been shown to be downregulated by the action of curcumin on the binding of nuclear proteins with interleukins or interferons [18].

The transcription factor AP-1 has been related to pro- and antiapoptotic effects in different types of cancer and its expression is downregulated by curcumin in in vitro models [19]. In microglial cells, curcumin was shown to inhibit the expression of (COX-2), while in melanoma cancer cells a concentration-dependent decrease in COX-2 was observed [20,21].

Curcumin has been found to modulate the cellular response of several cell types of the immune system, including B and T lymphocytes, macrophages and natural killer cells [22]. Curcumin can also affect both the expression and action of immune cytokines. TNF-a is a pleiotropic cytokine that induces growth stimulation and plays a pivotal role as an immunostimulant. Curcumin can modulate the expression of this factor and inhibit LPS-induced expression of TNF-α [23]. Dendritic cells, known for their immunostimulatory role, are also a target of curcumin. In particular, curcumin has been found to inhibit myeloid DC maturation, mainly through the suppression of CD80 and CD86 expression [24].

In some initial studies, curcumin supplementation showed no effect on the activity of natural killer (NK) cells in rats when given at doses of up to 40 mg/kg for up to five weeks [25]. However, in another study, Yadav et al. explored the immunomodulatory effects of curcumin and showed that this compound can increase NK cell cytotoxicity in vitro and that this effect can be further enhanced when curcumin was given along with IFN-γ treatment [26]. Increasing evidence indicates that curcumin exhibits its anti-inflammatory effects through the downregulation of NLRP3 inflammasomes [27]. Inflammasomes are multiprotein complexes that are responsible for innate immunity. The NLRP3, as a component of inflammasomes, can trigger immune response after detecting the products of damaged cells [28]. Curcumin can suppress NLRP3 inflammasome activation through regulation of the NF-κB signaling, leading also to suppression of IL-1β secretion [29]. For example, in malignant mesothelioma, curcumin has been found to exhibit its anticancer properties through regulation of signaling pathways affecting inflammasomes, such as IL-1 and NF-κB [30]. 

Other studies have also determined the anti-inflammatory and anticancer properties of curcumin. In a clinical investigation where human chronic myelogenous leukemia cells (K562) were studied, curcumin treatment resulted in significant downregulation of IL-6, TLRs, IL-3 and STAT-1 [31]. In another randomized double-blind placebo-controlled trial, 80 patients with solid tumors were randomly assigned to 180 mg/day of curcuminoids supplementation or matched placebo for 8 weeks. All mediators of inflammation, including interleukins, TNF-α and monocyte chemotactic protein-1 were significantly lower in the curcuminoids versus placebo group [32]. When curcumin was given as part of a botanical drug containing multiple polyphenols, named APG-157 in a double-blind, randomized phase 1 clinical trial, where 12 patients with oral cancer were enrolled, reduced IL-1β, IL-6, and IL-8 concentrations were found in the saliva 24 h after treatment [33].

## 3. Lung Cancer

Lung cancer is among the most commonly occurring types of cancer in men and the third most common in women. The standard treatment for lung cancer includes surgery, followed by radiation therapy, chemotherapy, and immunotherapy [34].

Curcumin exhibits its therapeutic potential in lung cancer treatment by acting on the Wnt/β-catenin-dependent pathway in the human lung cancer cell line A549 [35]. Curcumin also suppresses the vascular endothelial growth factor (VEGF0 and NF-κB expression in the same cell line [36]. In addition, curcumin has been shown to inhibit the expression of enhancer of zeste homolog 2 (EZH2) in lung cancer cells, which was followed by a decrease in the expression of the Notch 1 gene [37]. Curcumin has exhibited cytotoxicity against non-small-cell lung cancer (NSCLC) through inhibition of cell proliferation, increased apoptosis, and G_2_/M arrest. Curcumin also induces ROS production, thus activating the DNA damage signaling pathway [38].

A recent study investigated the effects of long-term, low-dose curcumin administration in NSCLC cells. Doses of curcumin between 0.25 and 0.5 μΜ produced significant suppression of both the invasion and metastatic potential of the cancer cells [39]. Curcumin derivatives have also shown promising results against the growth and invasion of NSCLC cells with anaplastic lymphoma kinas (ALK) rearrangements even when they become resistant to ALK inhibitory drugs [40].

The extracellular signal-regulated kinase (ERK) pathway plays an important role in the sensitivity of cancer cells to chemotherapeutic agents [41]. Curcumin selectively targets the ERK 1/2 pathway, resulting in a 75% reduction in its expression [42]. The phosphoinositide 3-kinase (PI3K)/Akt-dependent pathway has been related to cell proliferation and apoptosis in several cancer cell lines, but currently the available data on the relationship between the PI3K/Akt pathway and apoptosis are conflicting. Some drugs trigger apoptosis in cancer cells via stimulation of this pathway [43], while other drugs exert an inhibitory effect [44]. A recent study showed that a new derivative of curcumin, T59, activated the PI3K/Akt pathway and induced apoptosis in lung cancer cells [45], but another study found that a combination of curcumin and paris saponin 2 (PS2) induced apoptosis in lung cancer cells through inhibition of the PI3K-Akt pathway.

Tobacco smoke plays a significant role in the development of lung cancer. It has been estimated that approximately 90% of all lung cancer cases are associated with tobacco smoke [46]. The mitogen-activated protein kinase (MAPK) pathway is involved in the epithelial–mesenchymal transition (EMT) that is induced by tobacco smoke (TS). [47] Liang et al. investigated the effects of curcumin on the tobacco smoke-induced EMT in nude mice and concluded that curcumin can attenuate EMT alterations through downregulation of the MAPK/AP-1-dependent pathway [48]. In another study, Xie et al. investigated the possible effect of curcumin on TAp63α transcription factor (tumor suppressor gene). This factor is repressed by TS-induced lung cancer EMT, and it was found that curcumin can increase TAp63α expression and thus play a positive role in smocking-induced carcinogenesis [49].

## 4. Breast Cancer

Breast cancer continues to be the commonest type of cancer among women worldwide. In 2018, 2.1 million women were newly diagnosed with breast cancer and approximately 627,000 died from this disease [50]. The current clinical approach is based on the use of compounds targeting a shared pathway between different types of cancer cells in the same tumor [51]. For example, common pathways, including Notch, human epidermal growth factor receptor 2 (Her-2), NF-κB, and signal transducer and activator of transcription 3 (STAT-3), are targets of various treatmentoptions used against breast cancer [52].

The Akt/mTOR-dependent pathway is a leading signaling pathway in the proliferation of breast cancer cells, and clinical evidence suggests that targeting of this pathway is a promising treatment option [53,54]. Curcumin was found to inhibit the phosphorylation of Akt, mTOR and their downstream proteins, resulting in cell cycle arrest of various breast cancer cell lines, including T47D and MCF7 [55].

NF-κB plays a key role in the proliferation, invasion, and metastasis of breast cancer cell lines. Curcumin was found to suppress the nuclear translocation of NF-κB and to downregulate the levels of p100 and p52 in MCF-7 and MDA-MB-453 breast cancer cells, respectively [56]. Curcumin was also observed to induce cell death in MCF-7 breast cancer cells via upregulation of the expression of the spermidine/spermine N1-acetyltransferase (SSAT) gene, which is strongly related to the NF-κB-dependent signaling pathway [57].

The autocrine growth hormone (GH) signaling pathway may promote breast cancer proliferation by inducing abnormal cell growth, metastasis, and drug resistance. Curcumin treatment prevented GH-mediated invasion and metastasis in T47D breast cancer cells through suppression of the miR-182-96-183 cluster expression and also induced downregulation in antiapoptotic proteins, including Bcl-2 and Bcl- xL in the same cell line [58].

Curcumin has shown promising results in counteracting the drug resistance commonly found in various types of cancer, including breast cancer. For example, Flap endonuclease 1 (FEN1) overexpression in breast cancer cell lines promotes resistance to the chemotherapeutic agent cisplatin. Curcumin was found to enhance the sensitivity of breast cancer cells to cisplatin by downregulating FEN1 expression in vitro [59]. Paclitaxel, another significant chemotherapeutic agent used in the treatment of breast cancer, has demonstrated acquired resistance in breast cancer cell lines, possibly through the overexpression of the multidrug resistance mutation 1 (MDR-1) gene and P-glycoprotein. Curcumin has shown promising results in decreasing this drug resistance by downregulating MDR-1 gene expression in MCF-7 breast cancer cells [60].

## 5. Prostate Cancer

Prostate cancer is currently the second cause of cancer death among men in the Western world [6]. The initiation and progression of the disease are primarily driven by androgen receptor (AR)-dependent signaling [61]. In recent years, clinical practice has been focused on the use of AR pathway inhibitors (ARPIs), such as abiraterone and enzalutamide, for the treatment of both metastatic and non-metastatic prostate cancer. Because various mechanisms of treatment resistance have emerged, new potent agents are being investigated as possible treatment options [62].

Several in vitro studies have identified curcumin as an effective agent in the treatment of prostate cancer. Curcumin interferes with various molecular signaling pathways, including the NF-κB, mitogen-activated protein kinase (MAPK) and epidermal growth factor (EGFR) pathways [63]. Abnormal NF-κB activity has been observed in human prostate cancer cells and xenografts [64]. During prostate carcinogenesis, the NF-κB pathway induces cancer cell survival, angiogenesis and metastasis, but also chemoresistance to several agents. Curcumin, acts on this pathway and prevents the NF-κB activation, thus downregulating cancer-related genes, including Bcl-2, Bcl-xL, IL-6, and COX-2 [65]. Moreover, curcumin downregulates C-X-C motif chemokine ligand 1 (CXCL-1) and CXCL-2 by targeting the NF-κB pathway in androgen independent prostate cancer (AIPC) cells [66].

In addition to these properties, curcumin has demonstrated the ability to suppress AR gene transcription in prostate cancer cells in vitro downregulating AR expression in the lymph node carcinoma of the prostate (LNCaP) cell line by inhibition of the expression of steroidogenic acute regulatory proteins, such as HSD3B2 and CYP11A1 [67]. AP-1 is a transcription factor that is primarily activated by MAPKs. The activation of this transcription factor in prostate cancer cells often leads to a poor clinical outcome [68]. Curcumin significantly hampers AP-1 protein in AIPC (PC-3) cells, thus suppressing tumor progression. A recent study used the LNCaP cell line to investigate the effects of curcumin in the JNK-dependent signaling pathway. Under curcumin treatment, JNK levels were reduced, as were the levels of epigenetic markers, including H3K4. These findings suggest that curcumin has the capability to affect the transcriptional regulation of genes by regulating the expression of epigenetic markers [69].

A variety of studies have examined the effects of curcumin in androgen-insensitive prostate cancer cells. Dorai et al. found that curcumin treatment results in a 60–80% inhibition of PC3-insensitive prostate cancer cell line development [70], whereas Deeb et al. found out that curcumin decreased viability of both PC3 and DU-145 prostate cancer cells in vitro [71]. Interestingly, in castration-resistant prostate cancer (CRPC), where patients are no longer responsive to androgen deprivation therapy, curcumin seems to play a significant role in preventing cancer development as well. Yang et al. reported that curcumin can induce apoptosis and protective autophagy in CRPC cells, and that this effect is closely related to its iron-chelating properties. [72] Chen et al. investigated the effects of different curcumin analogues in the treatment of castrate-resistant prostate cancer. They found that some of the analogues induced apoptosis and inhibited the expression of nuclear factor (NF)-κB, AKT and p-AKT and thus they are considered potent anticancer agents against this type of prostate cancer [73].

## 6. Brain Tumors

Several brain malignancies are among the most resistant tumors to all therapeutic modalities [74]. Glioblastoma (GBM) is the most common primary malignant central nervous system (CNS) tumor, accounting for 45.6% of primary malignant brain tumors [75]. Although curcumin has shown poor bioavailability due to its rapid metabolism, low stability and poor permeability to the BBB, in a free form [76], localized delivery of curcumin into brain with the use of nanoparticles has significantly increased its delivery into the targeted brain nuclei [77]. The effect of curcumin on glioma cells has been evaluated in vitro and found to possess inhibitory effects, which is enhanced by miR-378 [78]. MiR-378 belongs to a class of non-coding RNAs closely associated with post-transcriptional gene regulation and it is expressed at lower levels in different brain tumor tissues. In addition, curcumin decreases the proliferation of glioblastoma cells in vitro via inhibition of miR-21 which is a significant regulator in GBM progression [79].

GBM stem cells are a small population of tumor cells responsible for tumor progression, recurrence, and resistance to chemotherapeutic agents [80]. Curcumin affects both the differentiation and self-renewal of these stem cells, resulting in the prevention of GBM recurrence [81]. Curcumin causes an arrest in GBM stem cells by inhibition of both the STAT3 and the IAP-dependent pathway, along with activation of the MAPK pathway. Thus, curcumin can effectively decrease the malignant characteristics of GBM stem cells [82].

EGFR overexpression has been associated with several types of cancer, including malignant brain tumors. The overexpression of EGFR is closely related to several molecular pathways, including the JAK/STAT-dependent and PI3K/Akt-dependent pathways. [83] In several studies, curcumin caused significant dose-dependent antiproliferative effects on different glioblastoma cell lines in vitro, by inhibiting the overexpression of EGFR [84,85,86]. Bojko et al. reported the ability of curcumin to treat human GBM cells in combination with EGFR kinase inhibitors, such as tyrphostin AG1478. Curcumin induced the cytotoxic potential of AG1478 and when given together, those two compounds resulted in irreversible DNA damage and a significant decrease in the cell viability of GBM cells [87].

## 7. Pancreatic Cancer

Pancreatic cancer is a fatal condition and it accounts for 3% of all cancers. Its highly heterogeneous nature and high rates of metastasis make this type of malignancy difficult to treat despite novel treatment options [88,89]. The effect of curcumin has been studied, along with other phytochemicals, in the treatment of pancreatic cancer since it can effectively suppress different metastatic properties of cancer cells. Early studies revealed the ability of curcumin to inhibit both the phosphorylation of extracellular signal-related kinases and platelet-derived growth factor, thus resulting in the inhibition of pancreatic stellate cells (PSCs) [90]. More recent studies explored the ability of curcumin to suppress the formation of cancer stem cells (CSCs) which are responsible for the high proliferation rate and rapid tumor growth of pancreatic cancer with promising results in suppressing tumor growth [91,92].

Several pancreatic cell lines are susceptible to the effects of curcumin, with Panc-1, BXPC-3, and L3.6pl being the most well-studied. In 2015, Zhao et al. revealed that curcumin can induce apoptosis in various pancreatic cancer cells by inhibition of the PI3 K/Akt pathway and induction of forkhead box O1 [93]. Osterman et al. demonstrated the antitumor effects of curcumin in panc-1 cells through decreasing both IAP protein and mRNA expression [94]. Cell division cycle 20 (Cdc20) plays an important oncogenic role in the growth of pancreatic cancer [95] and higher expression of Cdc20 is closely related to a poor prognosis [96]. Curcumin can inhibit Cdc20 expression in PC cells through upregulation of both Bcl-2-like protein 11 (Bim) and p21, leading to cell apoptosis and decreased cell motility [97].

## 8. Gastric Cancer

Gastric cancer is the third most common cause of cancer-related death worldwide [98]. The pathogenesis and growth of gastric cancer are associated with several signaling molecules, genes and epigenetic patterns [99]. Recent evidence indicates promising anticancer effects of curcumin via its ability to inhibit several signal transduction pathways, such as the p53, Ras, Wnt-β, extracellular signal-regulated kinases (ERK), PI3K, MAPKs and protein kinase B (Akt) in gastric cancer cells [100].

Another mechanism of in vitro gastric cancer cell growth inhibition of curcumin is by downregulation of nuclear transcription factors such as NF-κB and reduced expression of pro-inflammatory cytokines, including TNF-α, interleukins and chemokines [101]. Moreover, it can induce p53 gene expression, promoting apoptosis of the malignant cells [102]. Jin et al. reported that curcumin can increase p53 gene expression with subsequent inhibition of hepatic stellate cell growth [103]. Based on this p53 effect, Fu and colleagues found that curcumin may indeed induce apoptosis in gastric cancer cells by activation of the p53-dependent signaling pathway [104].

Several studies have explored the role of the phosphatidylinositol-3 kinase (PI3K)-dependent signaling pathway on cell proliferation in different types of cancer [105]. Fu et al. reported a close relationship between the effects of curcumin on gastric cancer and the PI3K pathway in two gastric cancer cell lines, SGC-7901 and BGC-823 [104]. In addition, a recent study conducted by Liu et al. indicated that curcumin can inhibit the proliferation of the SGC7901, MKN45 and NCI N87 cells lines through inhibition of the Wnt3 a/β-catenin/EMT pathway, along with regulation of the Bcl-2 signaling and caspase pathways [106].

## 9. Leukemia

Leukemia accounts for 8% of all malignancies worldwide [107]. It is the most common type of childhood cancer, accounting for 30% of cancer in children [108]. Leukemia has been categorized in four main types: chronic myeloid leukemia (CML), acute myeloid leukemia (AML), chronic lymphocytic leukemia (CLL) and acute lymphoblastic leukemia (ALL) [109].

The pathogenesis of CML is based on the expression of P210 BCR-ABL protein, encoded by the Breakpoint Cluster Region-Abelson (BCR-ABL) gene. This protein takes part in the proliferation of progenitor cells through its connection with pathways such as the Ras/Raf/MAPK pathway [110]. Curcumin has been identified as a potent agent against CML through downregulation of p210 BCR-ABL, resulting in inhibition of the MAPK pathway [111]. In addition, curcumin increased the efficacy of the chemotherapeutic agent imatinib, through downregulation of both the p210 BCR-ABL protein and the heat shock protein 90 (Hsp90) [112]. NF-κB, a transcription factor responsible for the regulation of different genes [113], is inhibited by curcumin in KCL-22 myeloid cells, resulting in apoptosis. Curcumin also enhanced the anticancer properties of-TNFα-related apoptosis-inducing ligand (TRAIL) in the same cell line since both compounds effectively downregulate the NF-kB-dependent pathway [15].

AML is characterized by poor prognosis and resistance to chemotherapy [114]. The presence of CD34+ AML cells are partly responsible for the limited anticancer effects of chemotherapeutic agents. Rao et al. explored the effects of curcumin on CD34+ AML cell lines, and specifically KG1a and Kasumi-1 when given in combination with daunorubicin. Curcumin was shown to enhance the cytotoxic effects of daunorubicin and to suppress the expression of Bcl-2 protein [115]. FMS-like tyrosine kinase 3 (FLT3) is a well-known marker in AML and its overexpression has been found in other hematological cancers, including ALL. Tima and colleagues reported a dose-dependent effect of curcumin on the expression of this biomarker, suggesting that curcumin can decrease the levels of both FLT3 and STAT5A in AML cells [116]. Zhu et al. explored the molecular mechanism of action of curcumin on human M5 leukemia cells (SHI-1 cell line) and reported that this compound can inhibit the MAPK and matrix metalloproteinase (MMP)-dependent signaling pathways as a result of its anticancer effects [117].

CLL is currently the commonest hematological malignancy in the Western world [118]. The disease is characterized bydefective neoplastic B lymphocytes and elevated T cells and natural killer (NK) cells [119]. Curcumin is a potent agent against CLL since it can suppress pathways involved in the growth and survival of neoplastic B lymphocytes. Ghosh et al. found that curcumin inhibited the STAT3, AKT, and NF-κB-dependent pathways in vitro, and suppressed the expression of the X-linked inhibitor of apoptosis protein (XIAP) and Mcl-1 [120].

ALL is characterized by overexpression of adenosine diphosphate ribose (ADP-ribose) and polymerase-1 (PARP1) [121]. Curcumin has been reported to be a potent agent against ALL via cleavage of PARP1-dependent pathways [122]. It may also affect other signaling pathways associated with the development and growth of ALL tumor, including AKT/mTOR, STAT5, and RAF/MEK/ERK. Curcumin reduced the activation of both the AKT/mTOR- and ABL/STAT5-dependent pathways in vitro and downregulated the expression of BCR/ABL. The ability of curcumin to affect those pathways explains its synergistic antitumor effects when given along with imatinib in the treatment of ALL [123]. The effects of curcumin on different cell signaling pathways in different cancers are summarized in Table 1.

## 10. Clinical Trials

Curcumin has been investigated, or is currently under investigation, both as a monotherapy and in combination with other drugs in various clinical trials. In a phase 2 clinical trial, curcumin was used alone in 21 patients with advanced pancreatic cancer. After receiving 8 g curcumin orally daily for 8 weeks, two patients showed clinical evidence of biological activity. Specifically, one patient had ongoing stable disease for >18 months and the other experienced brief, but significant tumor regression (73%) [124]. In another phase 1 clinical trial, 14 patients with advanced or metastatic breast cancer were given a combination of docetaxel and curcumin. Patients received 100 mg/m^2^ of docetaxel as a 1 h i.v. infusion and doses of curcumin ranging from 500 mg/day until dose-limiting toxicity occurred. Patients received a combination of a standard dose of docetaxel and different doses of curcumin for 7 consecutive days. It was observed that a dose of 6g/day of curcumin in combination with a standard dose of docetaxel was effective and safe against advanced breast cancer [125].

Another phase I/II study investigated the efficacy and safety of oral administration of curcumin alone or in combination with bioperine in 29 patients with multiple myeloma (MM) Patients received escalated doses of curcumin (2, 4, 6, 8, or 12 g/day in 2 divided doses) alone or with 10 mg/day of bioperine for 12 weeks. Based on the expression of surrogate biomarkers, including NF-kB (p65), COX-2 and phospho-STAT3, the mixed treatment was more effective than bioperine alone and curcumin was not associated with any toxic effects [126].

In patients with advanced breast cancer, curcumin is under investigation as monotherapy (NCT03980509) or in combination with paclitaxel (NCT03072992). The main object of these clinical studies is to determine the effect of curcumin on the development of advanced breast cancer and to estimate the risk of adverse effects.

The use of curcumin in patients with low-risk, localized prostate cancer under active surveillance will be evaluated with the goal to slowing cancer progression (NCT03769766). The possible radio- sensitizing effect of curcumin in prostate patients undergoing radiotherapy, along with the role of curcumin as a radio-protector in normal tissues will also be explored (NCT02724618).

In 2019, the combination treatment of curcumin and Avastin was given to patients with colorectal cancer with unresectable metastasis with the aim of evaluating progression-free survival, and overall survival (NCT02439385).

The efficacy and safety of curcumin in patients with advanced cervical cancer will be investigated in a Phase 2 clinical trial in which the treatment response rate will be a major indicator of efficacy and the Common Terminology Criteria for Adverse Events (CTCAE) classification will be used as an index of the safety of the therapeutic scheme (NCT04294836).

The tolerability of a combination of curcumin and cholecalciferol in the treatment of patients with untreated stage 0–II small lymphocytic lymphoma (SLL) or CLL was evaluated in a phase 2 clinical trial. When given together, the two drugs suppressed the growth of cancer cells and increased the overall survival rate (NCT02100423).

Some of the most recent clinical trials on the use of curcumin as a therapeutic agent alone or in combination with chemotherapeutic agents in various cancers are reported in Table 2.

## 11. Curcumin and Cancer-Associated Fibroblasts/Tumor-Associated Fibroblasts (CAFs/TAFs)

Fibroblasts are significant components of connective tissue [127]. Both cancer-associated fibroblasts and tumor-associated fibroblasts (CAFs and TAFs, respectively) have been shown to induce tumor growth and development and are currently considered primary source of different cytokines and tumor-promoting growth factors [128].

Curcumin can inhibit the development of fibroblasts in a dose-dependent way. Zhang and colleagues studied the effect of curcumin in CAFs isolated from human oral mucosa and found that curcumin increased the expression of Bax and similar pro-apoptotic molecules [129]. Other studies have shown that curcumin can inhibit several molecules involved in the TGF-β-pathway-induced fibrosis, including NF-Kb and the p38 MAP kinase [130] and increase the expression of a variety of tumor-suppressor proteins, such as p21 and p53, thus leading to inhibition of tumor invasion and cell migration [131].

On prostate-CAFs, curcumin can increase the intracellular ROS levels and induce G2/M arrest [132], while in tongue squamous cell carcinoma, curcumin has been found to inhibit the secretion of pro-carcinogenic cytokines, including stromal cell-derived factor-1 (SDF-1) and matrix metalloproteinases 2 (MMP2) [133].

## 12. Potential Side Effects of Curcumin

Curcumin has been characterized as “generally safe” by the US Food and Drug Administration (FDA) [134]. Indeed, no significant side effects related to curcumin can be found in the literature. Some of the documented cases are of reversible side effects, including allergic dermatitis [135]. Dose-escalating studies have demonstrated that consumption of up to 12 g of curcumin daily presents no damaging effects [136]. In studies on patients with solid tumors, no adverse effects were reported when curcumin was given for 8 weeks at a dosage of 900 mg/day orally, except for mild gastrointestinal upset [137]. Oral intake of 6 g/day of curcumin for 7 weeks was also reported to be safe in patients with breast cancer, and 3 g/day of curcumin given for 9 weeks to patients with prostate cancer showed no adverse effects [138,139].

Curcumin also exhibits a strong iron-chelating activity [140]. Long-term supplementation with curcumin induced iron depletion in young mice and this effect was further enhanced when they were fed with diets containing low iron concentrations [141]. Apart from that, curcumin possesses anticoagulant properties and may increase bleeding time in patients receiving anticoagulants [142].

Despite the absence of overt adverse effects of curcumin, it has been reported that this compound can inhibit several cytochrome P450 subtypes, including CYP2C9 and CYP3A4 [143,144]. For this reason, curcumin has been known to interact with certain other medications, including anticoagulants, antibiotics and antidepressants. For example, curcumin was found to affect the pharmacokinetics of warfarin and clopidogrel in Wistar rats and to increase the elimination half-life and volume of distribution of norfloxacin in New Zealand white rabbits [145,146].

## 13. Curcumin as Chemoprotective Agent in Cancer Chemotherapy

Curcumin has been found to inhibit some of the most significant chemotherapy-induced side effects. When given in combination with cisplatin, curcumin increased the levels of superoxide dismutase (SOD), an enzyme responsible for hepatoprotection [147]. In doxorubicin-treated cells, curcumin was found to upregulate the expression of SOD, and downregulate the expression of cardiotoxic marker SCK [148]. In cisplatin-treated cells, curcumin managed to diminish nephrotoxicity mainly through the downregulation of creatinine expression [149].

Interestingly, when a lecithin delivery system of curcumin was evaluated in a study containing 160 patients with solid tumors, in order to determine its efficacy in alleviating the side effects of chemotherapeutic drugs, results showed that curcumin can prevent the appearance of side effects related to cytostatic agents. That effect was mainly attributed to the ability of curcumin to downregulate inflammatory pathways [150].

## 14. Curcumin Application: Limitations and Prospects

Despite its efficacy and safety, curcumin displays poor solubility in water, a lowly pharmacokinetic profile and serious instability. For those reasons, the therapeutic potential of this compound is still under review. Even when administrated at doses of 12 g/day, the bioavailability of curcumin remains exceedingly poor [76]. Moreover, its oral bioavailability is low due to its low absorption by small intestine, extensive phase I and II biotransformation and quick elimination through the gall bladder [151].

A series of studies have been conducted to assess curcumin absorption after oral administration. Lao et al. performed a dose escalation study in which 24 healthy volunteers were given escalating doses of curcumin, ranging from 500 to 12,000 mg. Curcumin was not detectable in the serum of those participants when given at a dosage lower than 8 g [136]. In another study where healthy volunteers (*n* = 12) were also used, curcumin was administered at doses of 10 or 12 g. After 30 min, free curcumin could be detected in the plasma of only one volunteer [152]. The bioavailability of oral curcumin was also examined in patients with pancreatic cancer, where 21 patients received 8 g curcumin orally in combination with the chemotherapeutic agent gemcitabine. Total curcumin levels were found at a range of 29 to 91 ng/mL [153].

Another study on refractory colorectal cancer included 15 patients who were given daily curcumin doses ranging from 0.45 and 3.6 g for up to 4 months. Curcumin as well as its glucuronide and sulfate metabolites were detected in plasma samples after a dose of 3.6 g/day at the following concentrations: 11.1 ± 0.6, 15.8 ± 0.9 and 8.9 ± 0.7, respectively [154]. Despite its poor oral bioavailability, curcumin can cross the blood–brain barrier (BBB) thanks to its lipophilic nature. However, only a few studies in murine models have been able to assess its brain concentration. In one study, mice were administered 50 mg/kg curcumin orally and displayed brain curcumin concentrations lower than the limit of detection even 120 min after administration. However, when 100 mg/kg curcumin was given via intraperitoneal injection, total concentration ranged between 4 and 5 µg/g tissue [155].

In order to overcome poor curcumin absorption, and low bioavailability, different strategies, mainly in the form of developing novel oral delivery systems, have been made. Those strategies aim to increase curcumin solubility, improve its pharmacokinetic profile and enhance cellular uptake [156].

A water-soluble curcumin formulation consisting of turmeric extract, cellulosic derivatives and a widely-used hydrophilic carrier was made and compared to standard curcumin treatment in healthy volunteers. This formulation managed to increase the solubility of curcumin considerably and thus provide a 46-fold increase in its oral absorption [157].

Curcumin in a lipid-based formulation containing nanostructured lipid carriers is another promising way of increasing the bioavailability of curcumin. When a solid lipid curcumin particle (SLCP) formulation was given in both healthy volunteers and late-stage osteosarcoma patients, plasma curcumin levels were detectable in both healthy individuals and osteosarcoma patients as opposed to unformulated curcumin [158].

In a single-blind crossover study, healthy volunteers were given 500 mg of either unformulated curcumin, or liquid micelles or micronized curcumin. Both liquid micelles and micronized powder showed a relatively higher curcumin plasma concentration compared to the unformulated compound (3228, 41.6 and 7.1 nmol/L, respectively) [159].

In another study, SBA mesoporous silica 15 was used in order to prepare inhalable curcumin and investigate its application in lung cancer. The therapeutic effect of this inhalation system was evaluated and it was confirmed that the inclusion of curcumin in this mesoporous material increased the compound’s bioavailability and had a certain inhibitory effect on a metastatic lung mouse model. In addition, curcumin loaded in SBA silica 15 was more effective in reducing the number of metastatic lung tumors in the lung mouse model compared to treatment with curcumin alone [160].

In order to increase curcumin bioavailability, it is also important to protect it from physical and chemical damage due to its unstable nature under neutral or alkaline conditions. A well-studied technique is based on the use of metallocomplexes of curcumin, including complexation of curcumin with divalent ions, such as Zn2+, and Cu2+ [161]. Zebib and colleagues, evaluated the stability of curcumin complexes with divalent ions when prepared in a glycerol/water solvent and found that its stability was indeed significantly enhanced in vitro compared to curcumin alone [162].

In another study aimed to increase curcumin physicochemical stability, Meng et al. used zein/carboxymethyl dextrin nanoparticles to encapsulate curcumin. The results showed that encapsulation of curcumin in zein/CMD nanoparticles increased its stability and delayed its release in simulated gastrointestinal fluids [163].

## 15. Conclusions

Many challenges accompany the research on new drugs for use against malignant diseases. Various natural products have attracted the notice of researchers as possible chemotherapeutic agents, thanks to their efficacy and safety. Curcumin, an active hydrophobic polyphenol extracted from the rhizomes of the plant *Curcuma longa*, has been investigated thoroughly and has exhibited an important role in the treatment of various health conditions, including several types of cancer. As reported in this review, curcumin targets multiple signaling pathways involved in the initiation, development, and growth of tumors. Growth factors, transcription factors, protein kinases, cytokines, and genes taking part in apoptosis are the molecular targets of curcumin, which appears to significantly affect the development of various different malignancies. However, further in vitro studies and clinical trials in humans are needed to determine the complete mechanism of action of this compound in each different type of cancer, while ensuring its safety for human use.

## Figures and Tables

**Table 1:** Effects of curcumin on cell signaling pathways in different types of cancer.

Cancer Type	Cell Signaling Pathways	Type of Effect	Used Model	Tested Dosage	References
Lung cancer	Wnt/β-catenin	Downregulation/inhibition	Human cell line A549	60 μM	[35]
	VEGF	Downregulation/inhibition	Nude mice	100 mg/kg	[36]
	NF-κB	Downregulation/inhibition	Nude mice	100 mg/kg	[36]
	NOTCH 1	Downregulation/inhibition	Human lung cancer cell lines	6 μΜ	[37]
	ERK ½	Downregulation/inhibition	Human NCI-H1975 line	10 ng/mL	[41]
Breast cancer	Akt/mTOR	Downregulation/inhibition	Human breast cell lines	10 or 30 μΜ	[55]
	NF-κB	Downregulation/inhibition	Human breast cell lines	20 or 25 μΜ	[56]
	Autocrine GH	Downregulation/inhibition	T47D human breast cells	20 μΜ	[58]
	Bcl-2 and Bcl- xL	Downregulation/inhibition	T47D human breast cells	20 μΜ	[58]
	MDR-1	Downregulation/inhibition	MCF-7 breast cancer cell line	1.3 μΜ	[60]
	FEN1	Downregulation/inhibition	MCF-7 breast cancer cell line	0–50 μmol/L	[59]
Brain cancer	STAT3	Downregulation/inhibition	Human GBM stem cells	25 μΜ	[82]
	IAP	Downregulation/inhibition	Human GBM stem cells	25 μΜ	[82]
	MAPK	Upregulation/activation	Human GBM stem cells	25 μΜ	[82]
Pancreatic cancer	Platelet-derived growth factor	Downregulation/inhibition	Rat pancreatic stellate cells	25 μΜ	[90]
	PI3 K/Akt	Downregulation/inhibition	Panc-1 human pancreatic cells	20 μΜ	[93]
	IAP	Downregulation/inhibition	PANC-1 human cells	10/50/100μΜ	[94]
	Cdc20	Downregulation/inhibition	Patu8988 and Panc-1 human cell lines	10 or 20 μΜ	[97]
Gastric cancer	PI3K	Downregulation/inhibition	Human SGC-7901 and BGC-823 cells	10/20/40 μΜ	[104]
	Wnt3 a/β-catenin/EMT	Downregulation/inhibition	Human gastric cell lines	20 μΜ	[106]
	BCL-2	Downregulation/inhibition	Human gastric cell lines	20 μΜ	[106]
Leukemia					
-CML	MAPK	Downregulation/inhibition	Human K562 cell line	5 or 10 mg/L	[111]
	Hsp90	Downregulation/inhibition	Human K562 cell line	30 μΜ	[112]
	p210 BCR-ABL	Downregulation/inhibition	Human K562 cell line	5 or 10 mg/L	[111]
AML	Bcl-2	Downregulation/inhibition	Primary human CD34+ AML cells	0–80 μΜ	[116]
	MAPK	Downregulation/inhibition	Human SHI-1 cells	6.25–25 μM	[117]
	MMP	Downregulation/inhibition	Human SHI-1 cells	6.25–25 μM	[117]
CLL	NF-κB	Downregulation/inhibition	Human CLL B cells	10–12.5 μΜ	[120]
	STAT3	Downregulation/inhibition	Human CLL B cells	10–12.5 μΜ	[120]
	AKT	Downregulation/inhibition	Human CLL B cells	10–12.5 μΜ	[120]
	XIAP	Downregulation/inhibition	Human CLL B cells	10–12.5 μΜ	[120]
	Mcl-1	Downregulation/inhibition	Human CLL B cells	10–12.5 μΜ	[120]
ALL	AKT/mTOR	Downregulation/inhibition	Human ALL cell lines	0–40 μΜ	[123]
	ABL/STAT5	Downregulation/inhibition	Human ALL cell lines	0–40 μΜ	[123]
	BCR/ABL	Downregulation/inhibition	Human ALL cell lines	0–40 μΜ	[123]

**Table 2:** The most recent clinical trials of curcumin in different cancers.

Cancer	Drug	Title	NCT	Phase	Estimated/Actual Completion Date
Breast	Curcumin	A „Window Trial” on Curcumin for Invasive Breast Cancer Primary Tumors	NCT03980509	1	30 December 2022
	Curcumin ^®^ (CUC-01) and Paclitaxel	‘’Curcumin” in Combination with Chemotherapy in Advanced Breast Cancer	NCT03072992	2	30 June 2019
Prostate	Curcumin	Trial of Curcumin to Prevent Progression of Low-risk Prostate Cancer Under Active Surveillance	NCT03769766	3	November 2026
	Curcumin and radiation	Nanocurcumin for Prostate Cancer Patients Undergoing Radiotherapy (RT)	NCT02724618	2	April 2022
Colorectal	Avastin/FOLFIRI and curcumin	Avastin/FOLFIRI in Combination with Curcumin in Colorectal Cancer Patients with Unresectable Metastasis	NCT02439385	2	1 August 2019
Cervical	Curcumin	Curcumin in Advanced Cervical Cancer	NCT04294836	2	31 December 2023
Chronic Lymphocytic Leukemia (CLL), Small Lymphocytic Lymphoma (SLL)	Curcumin and cholecalciferol	Curcumin and Cholecalciferol in Treating Patients With Previously Untreated Stage 0–II Chronic Lymphocytic Leukemia or Small Lymphocytic Lymphoma	NCT02100423	2	13 December 2018

## Data Availability

Not applicable.
